# Roles for Endothelial Cells in Dengue Virus Infection

**DOI:** 10.1155/2012/840654

**Published:** 2012-08-16

**Authors:** Nadine A. Dalrymple, Erich R. Mackow

**Affiliations:** Department of Molecular Genetics & Microbiology, Stony Brook University, Stony Brook, NY 11794-5222, USA

## Abstract

Dengue viruses cause two severe diseases that alter vascular fluid barrier functions, dengue hemorrhagic fever (DHF) and dengue shock syndrome (DSS). The endothelium is the primary fluid barrier of the vasculature and ultimately the effects of dengue virus infection that cause capillary leakage impact endothelial cell (EC) barrier functions. The ability of dengue virus to infect the endothelium provides a direct means for dengue to alter capillary permeability, permit virus replication, and induce responses that recruit immune cells to the endothelium. Recent studies focused on dengue virus infection of primary ECs have demonstrated that ECs are efficiently infected, rapidly produce viral progeny, and elicit immune enhancing cytokine responses that may contribute to pathogenesis. Furthermore, infected ECs have also been implicated in enhancing viremia and immunopathogenesis within murine dengue disease models. Thus dengue-infected ECs have the potential to directly contribute to immune enhancement, capillary permeability, viremia, and immune targeting of the endothelium. These effects implicate responses of the infected endothelium in dengue pathogenesis and rationalize therapeutic targeting of the endothelium and EC responses as a means of reducing the severity of dengue virus disease.

## 1. Introduction

Dengue viruses are transmitted by mosquitoes and infect ~50 million people annually with an additional 2.5 billion people at risk living in tropical areas [1–3]. Expanding mosquito habitats are increasing the range of dengue virus outbreaks and the occurrence of severe diseases with 5–30% mortality rates: dengue hemorrhagic fever (DHF) and dengue shock syndrome (DSS) [1–3]. The majority of patients are asymptomatic or display mild symptoms of dengue fever (DF) which include rapid onset of fever, viremia, headache, pain, and rash [4]. Patients with DHF and DSS display symptoms of DF in addition to increased edema, hemorrhage, thrombocytopenia, and shock [1–3]. Although patient progression to DHF and DSS is not fully understood [3, 5], antibody-dependent enhancement (ADE) of dengue infection increases the potential for DSS and DHF [3, 6, 7]. There are four dengue virus serotypes (types 1–4) and infection by one serotype predisposes individuals to more severe disease following a subsequent infection by a different dengue serotype. The circulation of serotype-specific cross-reactive antibodies or preexisting maternal antibodies may contribute to progression to DHF/DSS by facilitating viral infection of immune cells and eliciting cytokine and chemotactic immune responses. In a murine antibody dependent enhancement model of dengue disease it was observed that a dramatic increase in infected hepatic endothelial cells (ECs) coincides with the onset of severe disease [8] and suggests a role for the endothelium in an immune-enhanced disease process during dengue infection. 

The major target tissues for dengue virus infection have been difficult to determine but virus has been isolated from human blood, lymph node, bone marrow, liver, heart, and spleen [9–14]. Blood samples are more easily obtained from dengue patients than tissues and yield a wide array of information about cytokine responses elicited by dengue virus infection [1–3, 14–18]. While many of these cytokines are present in DF patients, the majority of them are increased during DHF. Overall, DHF responses include greater cytokine production, T- and B-cell activation, complement activation, and T-cell apoptosis [3]. Complement pathway activation and elevated levels of complement proteins C3, C3a, and C5a are significant in that they can direct opsonization, chemotaxis of mast and other immune cells, and direct the localized release of the vascular permeability factor histamine from mast cells [17, 19–23]. Importantly, cytokines and complement factor responses all act on the endothelium and alter normal fluid barrier functions of ECs.

The ability of dengue virus to infect immune, dendritic, and endothelial cells fosters a role for immune responses to act on the endothelium and increase capillary permeability [5, 24–29]. However, the redundant nature of capillary barrier functions suggests that permeability is likely to be multifactorial in nature with many factors working in concert to modulate EC responses and permeabilize the endothelium. Dengue infected ECs are observed in DHF/DSS patient autopsy samples and in murine dengue virus disease models [8, 9, 14, 30]. This suggests that dengue infected ECs may also contribute directly to pathogenesis by increasing viremia, secreting cytokines, modulating complement pathways, or transforming the endothelium into an immunologic target of cellular and humoral immune responses.

Plasma constituents contain factors secreted by an estimated ~10^13^ ECs present in the body, and autopsy samples and murine dengue disease models clearly demonstrate that vascular ECs are infected [8, 9, 30, 31]. The endothelium is the primary fluid barrier of the vasculature and dengue virus-induced responses resulting in edema or hemorrhagic disease ultimately cause changes in EC permeability. Unique EC receptors, adherens junctions, and signaling pathways respond to cytokines, permeability factors, immune complexes, clotting factors, and platelets, normally acting in concert to control vascular leakage [5, 32–36]. Virally induced changes in endothelial or immune cell responses have the potential to alter this orchestrated balance with pathologic consequences [5, 32–35]. However, very little is known about the role of dengue virus-infected ECs in disease or the kinetics, timing, and replication of dengue viruses within patient ECs. The inability to kinetically study the endothelium in dengue patients and the relative ease of assessing blood components has resulted in a focus on immune cells instead of ECs. Yet, the endothelium is the ultimate target of permeabilizing responses. Here, we discuss studies of dengue infected ECs and the potential for the dengue infected endothelium to contribute to dengue pathogenesis.

## 2. Human Responses to Dengue Virus Infection

DHF and DSS are severe manifestations of dengue virus infection that result in increased vascular permeability, hemorrhage, and shock [3]. The presence of preexisting antibodies to dengue virus predisposes patients to severe disease following infection by a second dengue serotype [3]. A myriad of responses are associated with dengue infection that may contribute to disease, but the pathogenic mechanisms that result in DHF and DSS remain ambiguous [1–3, 26]. One common element of the dengue disease process is that enhanced immune responses increase vascular permeability by acting on the endothelium. Although it is clear that immune cells and their responses contribute to pathogenesis, the endothelium, which regulates vascular leakage, has not been considered a significant component of DHF and DSS [2, 5, 34, 35, 37].

### 2.1. Patient Studies

The ability of dengue virus to infect human ECs has been documented in autopsy samples of the heart, liver, and lung [9, 14]. In a postmortem study, Jessie et al. reported dengue virus antigen in sinusoidal ECs in the liver as well as macrophages, lymphoid cells in the spleen, the vascular endothelium of the lung, monocytes within the blood, and kidney tubules [9]. Salgado et al. also demonstrated the presence of viral antigen in endothelial cells within the heart and small myocardial vessels of a patient postmortem [14]. Depressed myocardial function has been associated with hemorrhagic forms of dengue virus infection [38]. Although no dengue virus RNA was detected in these cells by *in situ* hybridization, viral antigen uptake was also not confirmed. Additionally, no morphological damage to the endothelium was observed that might explain vascular leakage through disruption of the endothelium. However, the presence of circulating ECs, EC markers (VCAM and ICAM), and increased von Willebrand factor antigen and procoagulants, specifically in DHF patients, has been reported in other cases [39, 40]. Nevertheless, autopsy samples do not take into account contributions of dengue virus infection of ECs at earlier time points that may contribute to viremia and immune enhancing responses. Kinetic analysis of the endothelium in patients is invasive and has not been addressed. In general, little clinical data has been obtained between viral inoculation and onset of fever and viremia [3]. Thus, findings that ECs are infected with dengue have been marginalized since immune enhancing responses are presumed to be derived solely from immune cells. However, a variety of mechanisms exist for ECs to elicit immune enhancing cytokine and complement responses that recruit immune cells to the endothelium or directly alter barrier functions of EC adherens junctions [3–5, 41]. Finding that ECs are infected in patients suggests a direct means by which the infected endothelium may be altered by dengue virus. Additionally, preexisting antibodies may target DV antigen within infected ECs, further contributing to immune-enhanced permeability deficits observed in DHF and DSS.

### 2.2. Patient Responses to Infection and Markers of DHF/DSS

A hallmark of severe dengue disease is the presence of elevated levels of cytokines and chemokines including IP-10, ITAC, IL-1*β*, IL-2, IL-6, IL-8, IL-10, IL-12, IL-13, TNF*α*, IFN*α*, IFN*γ*, MIF, RANTES, histamine, and complement proteins C3, C3a, and C5a within blood and tissues [1–3, 14–18]. In patients, complement activation and an increase in complement protein products correlate with the severity of disease [42–45]. In a study by Avirutnan et al., C5b-9 complexes, complement-activated membrane-attack complexes, and C3a were formed on dengue-infected ECs in the presence of antibody—containing immune serum, though they did not direct complement-mediated cell lysis [26]. C3a is an anaphylatoxin that recruits mast cells and directs histamine release that locally increases vascular permeability [17, 19–22]. Elevated C3a, C5a, and histamine have been associated with severe permeability deficits in dengue virus patients and in the development of DHF and DSS [2, 3, 17, 26, 42]. Their presence in the blood of patients with severe dengue disease is significant since these anaphylatoxins direct lysis of infected cells and mast cell degranulation, leading to histamine release.

Importantly, cytokines, chemokines, and complement-activating factors can all be secreted by and act on the endothelium, influencing EC regulation of fluid barrier function and vascular leakage [5, 32–35]. The ability of dengue virus to infect the endothelium intimates that additional mechanisms could contribute to vascular permeability deficits through both direct- and immune-enhanced disease processes. Dengue infected ECs may elicit chemokine and cytokine responses that further activate or recruit immune cells to the endothelium and preexisting dengue antibodies may target viral proteins displayed on infected endothelium. Recent analysis of primary EC transcriptional responses indicates that dengue virus strongly induces secretion of immune cell activating cytokines, chemokines, and complement factors that are likely to contribute to an immune enhanced disease process [46]. Since permeability is ultimately the result of responses that act on the endothelium, dengue infected ECs are key elements in DSS and DHF that must be considered more fully within animal and *in vitro* models.

### 2.3. Roles of NS1 and Cross-Reactive NS1 Antibody

Dengue proteins may also play critical roles in enhancing DV pathogenesis within ECs through a variety of mechanisms. In particular, the NS1 protein is uniquely expressed in three forms: cytosolic, cell-surfaced expressed, and secreted [47–49]. Soluble secreted NS1 is both highly abundant and highly antigenic [50]. Likewise, NS1 antibodies are also present in high quantities and have been shown to bind the surface of platelets and ECs [51]. Because ECs are in steady contact with blood, they are susceptible to enhanced immune cell targeting promoted by adherent cross-reactive NS1 antibodies [51–53]. This targeting, as well as intracellular signaling triggered by direct NS1 antibody binding, may contribute to tissue-specific endothelial dysfunction and vascular leakage through EC activation [5, 51–53]. However, despite the abundance of NS1 antibodies that could promote leakage, vascular permeability is transient and additional contributions of NS1 have been poorly explored within humans and mouse models. Secreted NS1 can also bind cellular heparan sulfate E present on primary liver and lung ECs [54] and, along with the cell surface form of NS1, may further recruit circulating antibodies and immune factors to dengue infected ECs [55, 56].

As a secreted protein, the dengue NS1 protein modulates classical complement activation by binding to the C4b binding protein, thereby inactivating C4b [57]. Thus, cell-surface expressed NS1 on ECs could serve as a platform for C4b inactivation and antagonize classical complement activation pathways. Secreted NS1 may similarly attenuate complement activation by binding C1s/proC1s and C4 in a complex that reduces C4 levels required for complement pathway activation [58]. Together, NS1 and NS1 antibodies form a potent combination within DHF and DSS patients capable of eliciting or regulating immune and complement responses that act on the endothelium and contribute to dengue pathogenesis [59]. Curiously, the alternative complement pathway activator complement factor B, transcriptionally induced in dengue-infected endothelial cells [46], may induce C3a- and C5a-directed chemotaxis and histamine release by bypassing NS1 complement regulation. Antibody targeting of factor D, which activates factor B through cleavage, inhibits complement and leukocyte activation in nonhuman primates and several therapeutics have been developed that antagonize C3a and C5a receptor binding [60–63]. These advances suggest that the alternative pathway may be a new potential target for therapeutically reducing the severity of DHF and DSS diseases. Additional barrier stabilizing effectors that target the endothelium may also be considered as a means of therapeutically reducing vascular leakage and inflammation that contribute to dengue pathogenesis [64, 65].

## 3. *In Vivo* Animal Models of Dengue Virus Infection

### 3.1. Mouse Models of Dengue Virus Infection

Progress in understanding dengue pathogenesis has been hampered by the lack of suitable mouse models that replicate human cellular tropism and disease symptoms. In normal mice, dengue infection results in limb paralysis and little mortality [31]. Recently mouse-adapted dengue strains that mimic aspects of severe human disease in interferon (IFN) receptor knockout AG129 mice have been used as a dengue virus animal model [8, 31, 66–68]. Organ damage, hemorrhage, vascular leakage, viremia, and elevated cytokine levels analogous to that in humans are observed following dengue infection of AG129 mice [66, 67]. In one study, high titer inoculation of mice initiated TNF*α*-induced apoptosis of ECs, leading to vascular leakage [69]. However, IFN defective murine models further complicate our understanding of the dengue disease process since they do not fully mimic human responses to infection and lack IFN responses that limit dengue spread and induce EC proliferation and repair. Despite these limitations, current models have provided new insight into dengue virus pathogenesis and allow for kinetic studies of dengue virus infection of ECs.

Vascular leakage occurs in AG129 mice infected with mouse-adapted dengue strains and several studies have isolated murine-infected ECs within the spleen and liver [8, 30]. In support of a role for infected ECs in mediating severe dengue disease, Zellweger et al. recently reported that in the presence of subneutralizing levels of dengue-specific antibodies (ADE-mediated infection), a large percentage of infected liver sinusoidal ECs (LSECs) were detected and correlated directly with disease severity [8]. No evidence for ADE-mediated infection of ECs exists *in vitro* [70], although liver sinusoidal cells reportedly express Fc*γ* receptors that may contribute to immune enhanced infection of liver ECs [71]. In the same study, infection of mucosal macrophages was not enhanced by the presence of dengue antibodies and occurred after detection of infected LSECs, suggesting that increased viral loads from LSEC infection, but not ADE, enhanced immune cell infection [8]. Although a mechanism for this occurrence has yet to be determined, these findings give importance to the role of ECs in mediating dengue pathogenesis in the mouse animal model. Therefore, in addition to increasing viremia, the ability of dengue virus to infect ECs *in vivo* may provide a means for infection to alter capillary permeability and induce cytokine responses from ECs that recruit immune cells and contribute to dengue pathogenesis.

### 3.2. Mouse Model Responses Influenced by IFN

Since IFN plays a significant role in the regulation of viral spread and the growth and repair of the endothelium [3, 72–76], it is important to consider the consequences of dengue infections occurring in IFN unresponsive mouse models. Since IFN reportedly stimulates EC proliferation [76], IFN secretion by dengue infected cells is also likely to contribute to vascular repair following dengue infection, and the absence of the IFN-signaling response may explain the enhanced pathogenesis of dengue infections in IFN receptor knockout AG129 mice [31, 67, 77]. This absence abrogates antiviral responses that may naturally curb infection [72, 74]. Likewise, the dengue NS5 protein, which interferes with downstream IFN signaling to permit virus replication through STAT2 degradation, is unable to bind mouse STAT2 [78, 79]. These differences cloud interpretation of results from dengue-infected mice and may in fact contribute significantly to hemorrhagic responses that may or may not reflect normal pathogenic mechanisms. Current work is ongoing to address the lack of IFN responses within mice and create knock-in mice, which harbor functional human STAT2 [79].

### 3.3. Nonhuman Primate Models

Limited studies have also been conducted on nonhuman primates (NHPs) as an animal model. However, NHPs display almost no human symptoms of DF/DSS/DHF despite detectable virus replication [80]. Gene profiling following infection in NHPs revealed a potent antiviral response yet, in contrast to humans, almost no production of type I or II interferons or inflammatory cytokines [81]. Thus, murine models still appear to be the most suitable animal model for studying dengue infection, specifically in relation to cellular tropism and EC responses. Further work continues to explore new mouse adaptations that may one day produce animal models that fully mimic human responses to dengue infection.

## 4. *In Vitro* Infection of Endothelial Cells

### 4.1. Use of Endothelial Cell Lines for Studying Dengue Virus Infection

The difficulty of analyzing infections of the endothelium *in vivo* has driven the *in vitro* exploration of dengue infection of ECs. *In vitro*, ECs from various sources are permissive for dengue virus infection and have been used to study pathophysiological changes occurring within the endothelium following dengue virus infection. However, not all EC lines are equivalent and this has led to confusing and often contradictory results [82, 83]. Cell lines derived from endothelial and epithelial cell fusions are not representative of primary ECs and the ECV304 endothelial cell line has been shown to be bladder carcinoma and not endothelial in nature [84]. However, early passage primary human ECs permit investigation of dengue virus infection that approximates the human endothelium for analysis of dengue-altered EC responses.

### 4.2. Replication and Receptors for Dengue Virus on ECs

Dengue virus replicates within HUVECs (human umbilical vein ECs), LSECs, HPMEC-ST1.6R cells (human pulmonary EC line), ECV304 (endothelial cell line), and HMEC-1 cells (human microvascular EC line) [26, 27, 70, 85–89]. Recent *in vitro* studies demonstrated that efficient infection of primary ECs by dengue virus occurs as a result of attachment to heparan sulfate-containing cell surface proteins (HSPGs) [88]. HSPGs, specifically heparan sulfate glycoproteins of syndecan 2, also mediate attachment of dengue virus to K562 monocytes [90]. Although more specific HSPGs on ECs still need to be defined, an EC receptor blockade has the potential to reduce viremia, immune targeting of dengue virus infected ECs, and dengue virus-induced changes in ECs that contribute to pathogenesis.

### 4.3. Responses Elicited by Dengue-Infected ECs *In Vitro*

Several studies have focused on changes in the levels of cellular molecules or markers of EC activation, including VCAM and ICAM [91, 92]. Dengue virus infection upregulates cell surface markers of EC activation which can trigger the expression and release of various cytokines, chemokines, and complement factors that act on neighboring tissues, ECs, and circulating immune cells. Analysis of dengue-induced permeability responses suggested that EC permeability was increased *in vitro* [93]. Although a productive infection was not verified in this study, permeability occurred in conjunction with a decrease in VE-cadherin, which regulates the fluid barrier function of adherens junctions [93, 94]. 

Additional studies examined the induction or secretion of cytokines following dengue virus infection of primary HUVECs and ECV304, LSEC-1, HMEC-1, or HPMEC-ST1.6R cell lines [26, 27, 37, 85, 87, 89, 95]. Dengue infection of the HPMEC-ST1.6R cell line increased IL-6 and IL-8 (6–8 days p.i.) as well as vascular endothelial cell growth factor (VEGF) [27, 95]. Other studies have also singled out RANTES, IL-6 and/or IL-8 as cytokines elicited by dengue-infected ECs [26, 37, 46, 87], thus promoting the endothelium as a source of potent chemotactic cytokines in DSS/DHF patients. Both IL-8 and RANTES are chemotactic agents that can increase vascular permeability by localized attraction of neutrophils [96, 97]. Analyses of transcriptional changes elicited in response to dengue infection also show small increases in additional cytokines and antiviral IFN-stimulated genes [27, 37, 46]. IFN is highly induced in dengue patients and these findings suggest that ECs are likely to contribute to circulating IFN responses.

### 4.4. Kinetics of Dengue Virus Infection *In Vitro*

Dengue infected endothelial cell lines reportedly induce a low level of infection (<10%) and coordinately low-level transcriptional responses [26, 85]. However, analysis of >90% uninfected cells at late time points after infection (3–8 days) makes it difficult to accurately assess the contribution from dengue infected cells. This is compounded by innate antiviral responses elicited by infecting a small number of ECs and the stimulation of IFN responses by >90% uninfected ECs.

A recent kinetic analysis of primary EC responses to dengue virus infection paints an important picture of the endothelium's role in dengue disease. Primary EC monolayers are ~80% infected by dengue virus and rapidly produce virus by 24 hours after infection [88]. Both the production of progeny virus and the number of infected ECs within monolayers decrease 2-3 days after infection [88]. Dengue virus titers following EC infection were nearly identical to viral titers observed in IFN-deficient VeroE6 cells [88], suggesting that virus regulates early cellular interferon responses of ECs. In fact, the lack of viral spread reported in some studies, where fewer than 10% of ECs were infected [37, 70], is consistent with the paracrine effect of interferon produced by a small number of initially infected cells. Thus, *in vitro* studies point to the infected endothelium as a likely source of viremia following dengue virus infection. These findings also highlight the importance of assessing the impact of EC infections at early times following *in vivo* infection, something more easily achieved within mouse models than patients.

Recovering DHF patients regain normal endothelial function quickly, implying a transient effect and early recovery of EC functions following infection [3]. A rapid productive infection of the endothelium is difficult to assess in humans, but *in vitro* studies show that infected ECs rapidly release virus. This suggests that dengue infected ECs may contribute to early viremia in dengue patients and present dengue virus antigens on EC surfaces that may be targeted by immune cells [88]. Infected ECs may also elicit cytokine and chemokine responses that can act directly on the endothelium [26, 27, 37, 85, 87, 89, 95]. Endpoint sampling of ECs at autopsy or analysis of endothelial function within recovering patients does not take into consideration an early or transient infection of ECs as is observed *in vitro*. The lack of apparent spread within EC monolayers and the apparent decrease in infected cells 2-3 days after infection [46] also suggest that EC-elicited IFN responses may limit dengue virus spread *in vitro* and contribute to high levels of circulating IFN in dengue patients. Interestingly, IFN treatment directs EC proliferation and may be a response that both limits viral spread and activates the EC repair process [76]. In fact, EC proliferation in response to IFN may explain the absence of endothelial damage within DHF patients and the presence of vascular leakage in IFN receptor deficient mouse models.

## 5. Conclusions

The endothelium is not a static channel that simply separates the vasculature from surrounding tissue [33–35]. The endothelium dynamically elicits responses that may contribute to immune enhancement and vascular permeability during dengue virus infection. Several hypotheses have been offered to explain the development of severe dengue disease and immune enhanced responses clearly impact barrier functions of the endothelium, but pathogenic mechanisms that result in DHF and DSS remain vague [1–3, 26]. Leakage of the vascular endothelium is a central component of dengue virus disease and studies discussed here suggest that the dengue infected endothelium may contribute to pathogenic immune responses and immune targeting of the endothelium. However, the role of dengue virus infected ECs in pathogenesis requires definitive *in vivo* kinetic studies which are difficult to perform in patients. The ability of the endothelium to respond to immune cells resulting in capillary permeability further highlights the importance of dengue infected ECs in pathogenesis. The central importance of the endothelium in dengue disease suggests that stabilizing fluid barrier functions of the endothelium may be a therapeutic approach for reducing vascular leakage in DHF and DSS patients [64, 65].
